# 6-Bromo-9,9-diethyl-*N*,*N*-di­phenyl­fluoren-2-amine

**DOI:** 10.1107/S2414314624011763

**Published:** 2024-12-20

**Authors:** Themmila Khamrang, A. Kannan, C. Ponraj, Madhukar Hemamalini, G. Jerald Maria Antony, Dhandayutham Saravanan

**Affiliations:** a Department of Chemistry Dhanamanjuri University, Manipur 795 001 India; b Department of Chemistry Anjalai Ammal Mahalingam Engineering College, Kovilvenni Tiruvarur 614 403 Tamil Nadu India; c Department of Chemistry, National College, Tiruchirappalli, Tamil Nadu, India; d Department of Chemistry Mother Teresa Women’s University, Kodaikanal Tamil Nadu India; University of Aberdeen, United Kingdom

**Keywords:** crystal structure, C—H⋯π inter­actions

## Abstract

In the title compound, C_29_H_26_BrN, the dihedral angles between the fluorene fused-ring system and the pendant phenyl groups are 67.76 (12) and 88.38 (12)°. In the crystal, weak pairwise C—H⋯π inter­actions link the mol­ecules into inversion dimers.

## Abstract 2

In the title compound, the dihedral angles between the fluorene fused-ring system and the pendant phenyl groups are 67.76 (12) and 88.38 (12)°. In the crystal, weak pairwise C—H⋯π inter­actions link the mol­ecules into inversion dimers.

## Graphical abstract

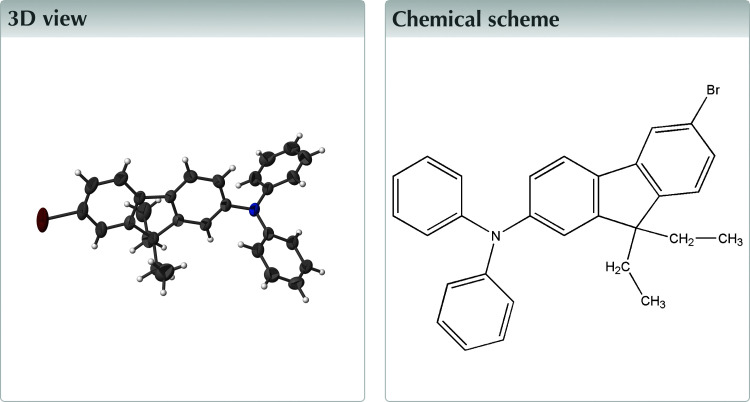

## Structure description

Some fluoren-9-imines show fluorescence properties (Dufresne *et al.*, 2011 ▸) and potential as organic components in materials with flexible HOMO–LUMO gaps (Eakins *et al.*, 2013 ▸). The crystal structures of *N*-mesityl-9*H*-fluoren-9-imine (Evans *et al.*, 2016 ▸), *N*-(4-chloro­phen­yl)-9*H*-fluoren-9-imine (Crundwell *et al.*, 2019 ▸), 9-(4-bromo­but­yl)-9*H*-fluorene-9-carb­oxy­lic acid (Zhang *et al.*, 2014 ▸) and 9,9-diethyl-9*H*-fluorene-2,4,7-tricarb­aldehyde (Seidel *et al.*, 2021 ▸) have been reported. As part of our research in this field, we present the synthesis and structural characterization of the title compound, C_29_H_26_BrN, (**I**).

**Figure 1:**
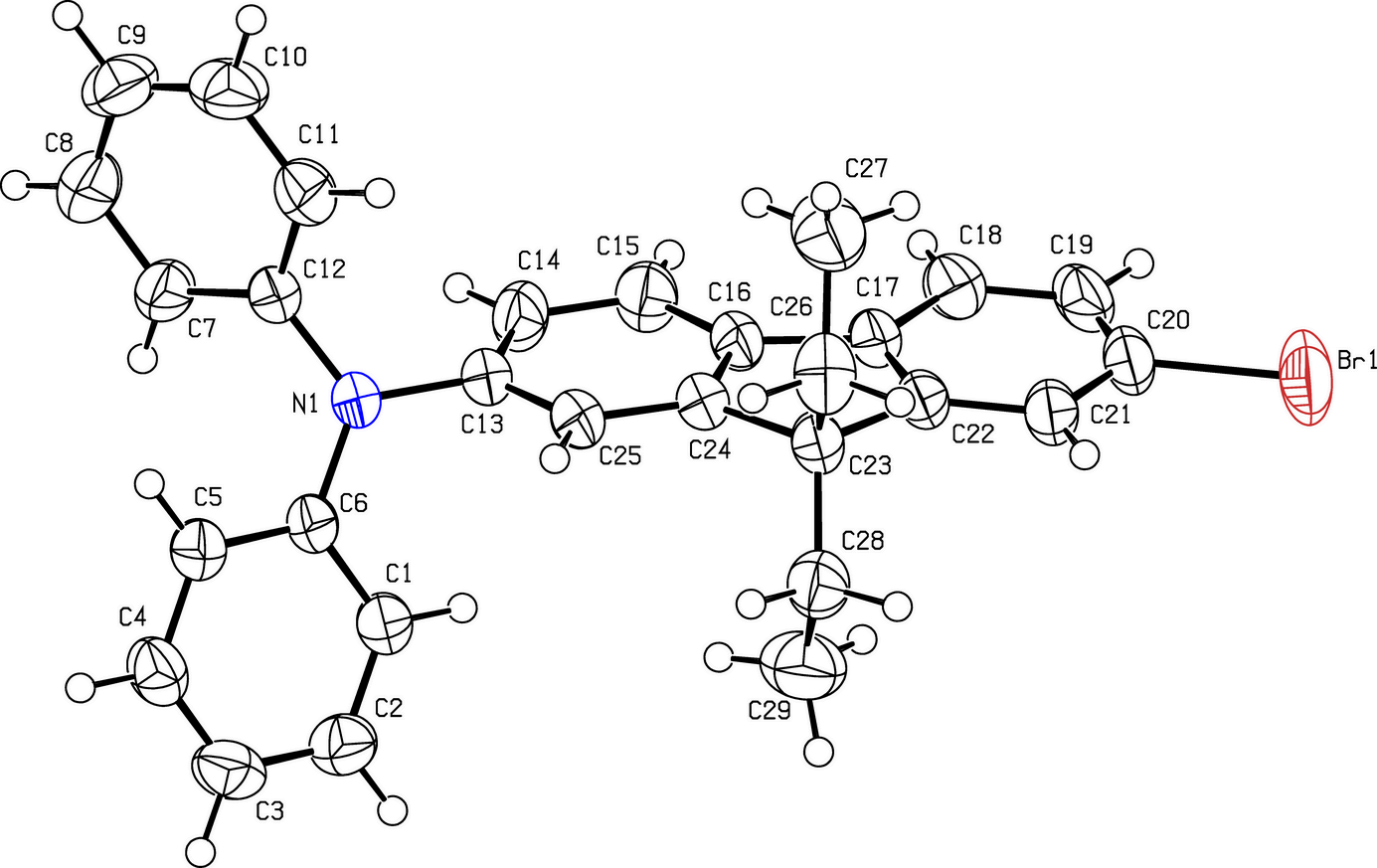
The asymmetric unit of (**I**). Displacement ellipsoids are drawn at the 50% probability level.

**Figure 2:**
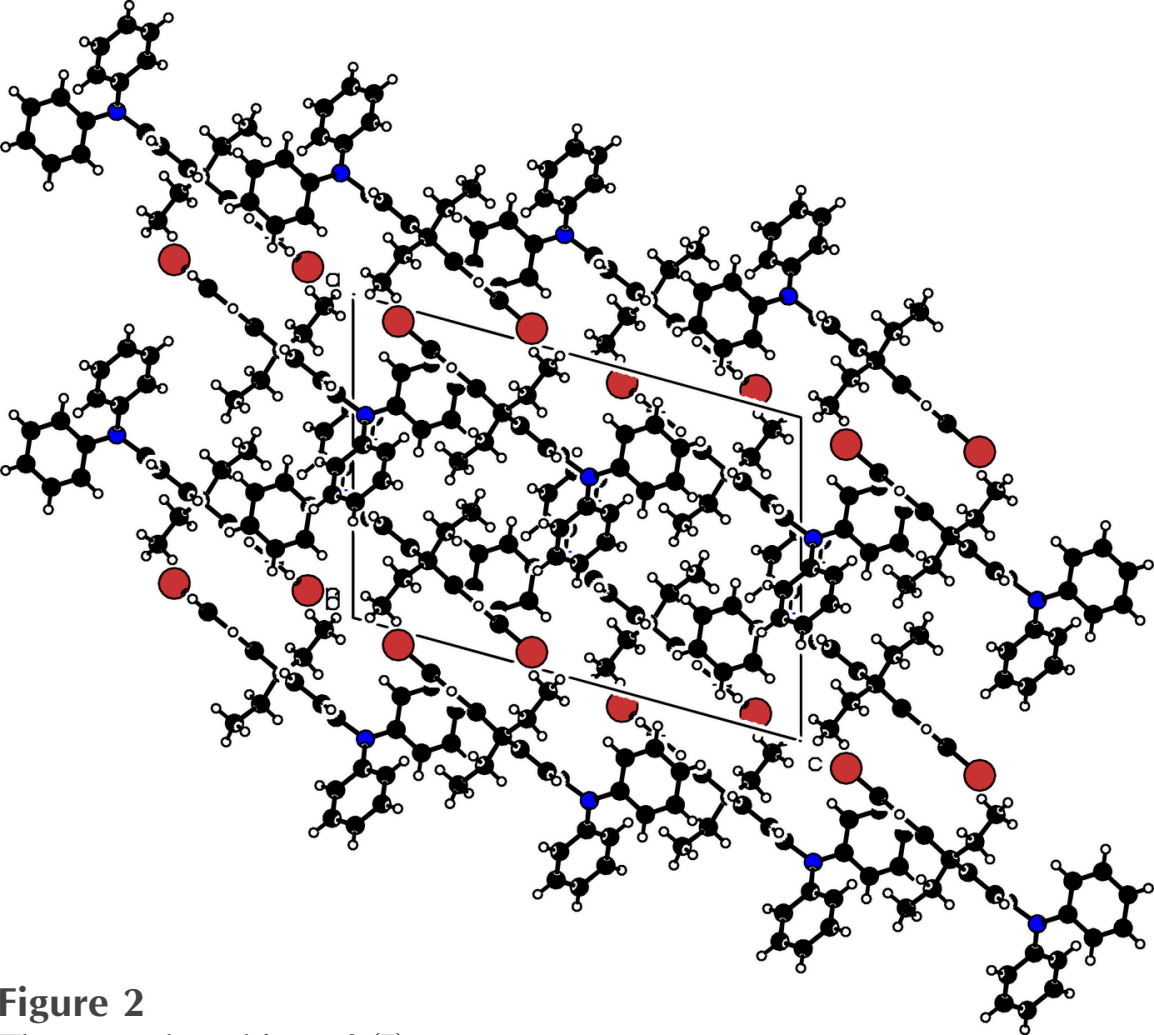
The crystal packing of (**I**).

The asymmetric unit of (**I**) contains one mol­ecule (Fig. 1 ▸) in space group *P*2_1_/*c*. The dihedral angles between the C13–C25 fluorene fused ring (r.m.s. deviation = 0.030 Å) and the pendant C1–C6 and C7–C12 phenyl groups are 67.76 (12) and 88.38 (12)°, respectively; the dihedral angle between the phenyl groups is 60.96 (16)°. The packing of the crystal structure is illustrated in Fig. 2 ▸. Neighboring mol­ecules within the structure are linked by pairwise C—H⋯π inter­actions, as detailed in Table 1 ▸.

A search of the Cambridge Structural Database (Version 5.43, update November 2022; Groom *et al.*, 2016 ▸) for the fluoren-9-imine fragment with additional substituents yielded 9*H*-fluoren-9-imine (CSD refcode EPAJEN: Kent *et al.*, 2021 ▸), *N*-[(2-nitro­phen­yl)sulfan­yl]-9*H*-fluoren-9-imine (REQXUI: Melen *et al.*, 2013 ▸), *N*-hy­droxy-9*H*-fluoren-9-imine (NIXWUO: Bugenhagen *et al.*, 2014 ▸), and *N,N′*-([1,1′-biphen­yl]-4,4′-di­yl)bis­(9*H*-fluoren-9-imine) (LODQEE: Sprachmann *et al.*, 2023 ▸).

## Synthesis and crystallization

The title compound was prepared by the literature method (Thomas *et al.*, 2005 ▸). Crystals suitable for X-ray diffraction were grown by recrystallization from di­chloro­methane solution.

## Refinement

Crystal data, data collection and structure refinement details are summarized in Table 2 ▸.

## Supplementary Material

Crystal structure: contains datablock(s) global, I. DOI: 10.1107/S2414314624011763/hb4495sup1.cif

Structure factors: contains datablock(s) I. DOI: 10.1107/S2414314624011763/hb4495Isup2.hkl

Supporting information file. DOI: 10.1107/S2414314624011763/hb4495Isup3.cml

CCDC reference: 2407284

Additional supporting information:  crystallographic information; 3D view; checkCIF report

## Figures and Tables

**Table 1:** Hydrogen-bond geometry (Å, °) *Cg*5 is the centroid of the C17–C22 ring.

*D*—H⋯*A*	*D*—H	H⋯*A*	*D*⋯*A*	*D*—H⋯*A*
C2—H19⋯*Cg*5^i^	0.93	2.87	3.692 (4)	148

Symmetry code: (i) 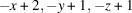.

**Table 2:** Experimental details

Crystal data	
Chemical formula	C_29_H_26_BrN
*M*_r_	468.42
Crystal system, space group	Monoclinic, *P*2_1_/*c*
Temperature (K)	293
*a*, *b*, *c* (Å)	12.7044 (14), 10.5123 (17), 18.2491 (19)
β (°)	105.372 (12)
*V* (Å^3^)	2350.0 (5)
*Z*	4
Radiation type	Mo *K*α
μ (mm^−1^)	1.77
Crystal size (mm)	0.37 × 0.32 × 0.29

Data collection	
Diffractometer	Agilent Xcalibur, Atlas, Gemini
Absorption correction	Multi-scan (*CrysAlis RED*; Agilent, 2012 ▸)
*T*_min_, *T*_max_	0.507, 0.578
No. of measured, independent and observed [*I* > 2σ(*I*)] reflections	8476, 4762, 2977
*R*_int_	0.029
(sin θ/λ)_max_ (Å^−1^)	0.625

Refinement	
*R*[*F*^2^ > 2σ(*F*^2^)], *wR*(*F*^2^), *S*	0.048, 0.117, 1.02
No. of reflections	4762
No. of parameters	282
H-atom treatment	H-atom parameters constrained
Δρ_max_, Δρ_min_ (e Å^−3^)	0.54, −0.60

Computer programs: *CrysAlis PRO* (Agilent, 2012 ▸), *SHELXT2018/2* (Sheldrick, 2015*a* ▸), *SHELXL2018/3* (Sheldrick, 2015*b* ▸) and *PLATON* (Spek, 2020 ▸).

## full crystallographic data

### 6-Bromo-9,9-diethyl-N,N-diphenylfluoren-2-amine Crystal data

**Table:**

C_29_H_26_BrN	*F*(000) = 968
*M**_r_* = 468.42	*D*_x_ = 1.324 Mg m^−^^3^
Monoclinic, *P*2_1_/*c*	Mo *K*α radiation, λ = 0.71073 Å
*a* = 12.7044 (14) Å	Cell parameters from 9567 reflections
*b* = 10.5123 (17) Å	θ = 3.6–26.5°
*c* = 18.2491 (19) Å	µ = 1.77 mm^−^^1^
β = 105.372 (12)°	*T* = 293 K
*V* = 2350.0 (5) Å^3^	Plate, colourless
*Z* = 4	0.37 × 0.32 × 0.28 mm

### 6-Bromo-9,9-diethyl-N,N-diphenylfluoren-2-amine Data collection

**Table:**

Agilent Xcalibur, Atlas, Gemini diffractometer	2977 reflections with *I* > 2σ(*I*)
Radiation source: fine-focus sealed tube	*R*_int_ = 0.029
ω scans	θ_max_ = 26.4°, θ_min_ = 3.5°
Absorption correction: multi-scan (CrysAlis RED; Agilent, 2012)	*h* = −9→15
*T*_min_ = 0.507, *T*_max_ = 0.578	*k* = −13→6
8476 measured reflections	*l* = −20→22
4762 independent reflections	

### 6-Bromo-9,9-diethyl-N,N-diphenylfluoren-2-amine Refinement

**Table:**

Refinement on *F*^2^	Primary atom site location: dual
Least-squares matrix: full	Hydrogen site location: inferred from neighbouring sites
*R*[*F*^2^ > 2σ(*F*^2^)] = 0.048	H-atom parameters constrained
*wR*(*F*^2^) = 0.117	*w* = 1/[σ^2^(*F*_o_^2^) + (0.0391*P*)^2^ + 1.125*P*] where *P* = (*F*_o_^2^ + 2*F*_c_^2^)/3
*S* = 1.02	(Δ/σ)_max_ = 0.001
4762 reflections	Δρ_max_ = 0.54 e Å^−^^3^
282 parameters	Δρ_min_ = −0.60 e Å^−^^3^
0 restraints	

### 6-Bromo-9,9-diethyl-N,N-diphenylfluoren-2-amine Special details

**Table:**

Geometry. All esds (except the esd in the dihedral angle between two l.s. planes) are estimated using the full covariance matrix. The cell esds are taken into account individually in the estimation of esds in distances, angles and torsion angles; correlations between esds in cell parameters are only used when they are defined by crystal symmetry. An approximate (isotropic) treatment of cell esds is used for estimating esds involving l.s. planes.
Refinement. All the H atoms were positioned geometrically (C—H = 0.93–0.97 Å) and were refined using a riding model, with *U*_iso_(H) = 1.2 *U*_eq_(C).

### 6-Bromo-9,9-diethyl-N,N-diphenylfluoren-2-amine Fractional atomic coordinates and isotropic or equivalent isotropic displacement parameters (Å^2^)

**Table:**

	*x*	*y*	*z*	*U*_iso_*/*U*_eq_	
Br1	0.95429 (3)	0.61601 (5)	0.10095 (2)	0.0858 (2)	
N1	0.6358 (2)	0.3480 (3)	0.52774 (13)	0.0483 (7)	
C6	0.6913 (2)	0.4055 (3)	0.59604 (15)	0.0397 (7)	
C12	0.5429 (2)	0.2704 (3)	0.52127 (15)	0.0403 (7)	
C5	0.6402 (2)	0.4278 (3)	0.65293 (16)	0.0457 (8)	
H16	0.567720	0.403893	0.646045	0.055*	
C25	0.6902 (2)	0.4777 (3)	0.43321 (15)	0.0425 (7)	
H2	0.668430	0.550728	0.453992	0.051*	
C1	0.7982 (2)	0.4448 (3)	0.60737 (16)	0.0462 (8)	
H20	0.833360	0.432118	0.569297	0.055*	
C24	0.7358 (2)	0.4858 (3)	0.37282 (15)	0.0389 (7)	
C16	0.7665 (2)	0.3769 (3)	0.34112 (15)	0.0417 (7)	
C4	0.6958 (3)	0.4849 (3)	0.71935 (17)	0.0510 (8)	
H17	0.660812	0.498929	0.757410	0.061*	
C22	0.8134 (2)	0.5466 (3)	0.27537 (15)	0.0415 (7)	
C17	0.8142 (2)	0.4145 (3)	0.27948 (16)	0.0429 (7)	
C23	0.7604 (2)	0.6046 (3)	0.33320 (15)	0.0420 (7)	
C21	0.8555 (2)	0.6077 (3)	0.22258 (16)	0.0489 (8)	
H6	0.856900	0.696032	0.220138	0.059*	
C7	0.5403 (2)	0.1756 (3)	0.57312 (17)	0.0502 (8)	
H22	0.600767	0.161627	0.614011	0.060*	
C13	0.6774 (2)	0.3589 (3)	0.46244 (16)	0.0439 (7)	
C26	0.6551 (3)	0.6756 (3)	0.29330 (19)	0.0572 (9)	
H30A	0.620478	0.704973	0.331615	0.069*	
H30B	0.674493	0.750156	0.268381	0.069*	
C11	0.4529 (3)	0.2877 (3)	0.46116 (17)	0.0525 (8)	
H26	0.453256	0.350736	0.425475	0.063*	
C3	0.8025 (3)	0.5218 (3)	0.73041 (19)	0.0550 (9)	
H18	0.840137	0.559872	0.775801	0.066*	
C14	0.7071 (3)	0.2511 (3)	0.43068 (17)	0.0538 (9)	
H13	0.697315	0.171879	0.450604	0.065*	
C2	0.8527 (3)	0.5018 (3)	0.67364 (18)	0.0530 (8)	
H19	0.924772	0.527292	0.680377	0.064*	
C15	0.7516 (3)	0.2588 (3)	0.36916 (18)	0.0534 (8)	
H12	0.770937	0.185547	0.347291	0.064*	
C28	0.8353 (3)	0.6960 (3)	0.38836 (18)	0.0554 (9)	
H28A	0.853799	0.765901	0.359389	0.066*	
H28B	0.795045	0.731182	0.421969	0.066*	
C18	0.8542 (3)	0.3425 (3)	0.22966 (17)	0.0538 (9)	
H9	0.853886	0.254087	0.232049	0.065*	
C20	0.8952 (2)	0.5344 (4)	0.17374 (16)	0.0534 (9)	
C19	0.8947 (3)	0.4046 (4)	0.17605 (17)	0.0594 (10)	
H8	0.921608	0.357949	0.141644	0.071*	
C10	0.3627 (3)	0.2128 (4)	0.4534 (2)	0.0614 (10)	
H25	0.302388	0.225092	0.412174	0.074*	
C8	0.4492 (3)	0.1026 (3)	0.5645 (2)	0.0616 (9)	
H23	0.448149	0.039306	0.599897	0.074*	
C9	0.3599 (3)	0.1205 (4)	0.5050 (2)	0.0631 (10)	
H24	0.297871	0.070585	0.499736	0.076*	
C27	0.5732 (3)	0.5986 (4)	0.2352 (2)	0.0750 (12)	
H31A	0.603564	0.576906	0.193923	0.112*	
H31B	0.507957	0.647711	0.216245	0.112*	
H31C	0.556058	0.522199	0.258433	0.112*	
C29	0.9385 (3)	0.6393 (4)	0.4357 (2)	0.0774 (12)	
H29A	0.974601	0.593977	0.403666	0.116*	
H29B	0.922103	0.581655	0.471999	0.116*	
H29C	0.985418	0.705712	0.462089	0.116*	

### 6-Bromo-9,9-diethyl-N,N-diphenylfluoren-2-amine Atomic displacement parameters (Å^2^)

**Table:**

	*U*^11^	*U*^22^	*U*^33^	*U*^12^	*U*^13^	*U*^23^
Br1	0.0712 (3)	0.1406 (5)	0.0560 (2)	−0.0011 (3)	0.03493 (19)	0.0268 (2)
N1	0.0527 (15)	0.0639 (18)	0.0314 (13)	−0.0212 (14)	0.0167 (11)	−0.0053 (12)
C6	0.0426 (16)	0.0447 (18)	0.0320 (14)	−0.0033 (15)	0.0100 (12)	0.0057 (14)
C12	0.0406 (15)	0.0489 (19)	0.0327 (15)	−0.0046 (15)	0.0121 (12)	−0.0013 (14)
C5	0.0433 (16)	0.056 (2)	0.0402 (16)	−0.0045 (16)	0.0147 (13)	0.0002 (15)
C25	0.0476 (17)	0.0478 (19)	0.0346 (15)	−0.0043 (16)	0.0155 (13)	−0.0045 (14)
C1	0.0435 (16)	0.058 (2)	0.0377 (16)	−0.0037 (16)	0.0123 (13)	0.0026 (15)
C24	0.0422 (16)	0.0428 (18)	0.0326 (14)	−0.0032 (15)	0.0114 (12)	0.0000 (14)
C16	0.0496 (17)	0.0450 (19)	0.0338 (15)	−0.0045 (16)	0.0167 (13)	−0.0039 (14)
C4	0.064 (2)	0.051 (2)	0.0419 (17)	0.0019 (18)	0.0213 (15)	−0.0049 (16)
C22	0.0375 (15)	0.056 (2)	0.0310 (14)	−0.0012 (16)	0.0099 (12)	0.0033 (14)
C17	0.0428 (16)	0.055 (2)	0.0339 (15)	−0.0055 (16)	0.0153 (13)	−0.0044 (15)
C23	0.0485 (17)	0.0449 (18)	0.0355 (15)	−0.0003 (16)	0.0162 (13)	0.0021 (14)
C21	0.0434 (16)	0.067 (2)	0.0377 (16)	−0.0026 (17)	0.0133 (13)	0.0083 (16)
C7	0.0452 (18)	0.056 (2)	0.0460 (18)	−0.0095 (17)	0.0069 (14)	0.0107 (16)
C13	0.0485 (17)	0.053 (2)	0.0332 (15)	−0.0129 (16)	0.0153 (13)	−0.0025 (14)
C26	0.058 (2)	0.060 (2)	0.058 (2)	0.0127 (19)	0.0254 (17)	0.0139 (18)
C11	0.0548 (19)	0.060 (2)	0.0405 (17)	−0.0001 (18)	0.0084 (15)	0.0087 (16)
C3	0.064 (2)	0.049 (2)	0.0468 (18)	−0.0053 (19)	0.0054 (16)	−0.0097 (16)
C14	0.074 (2)	0.0434 (19)	0.0515 (19)	−0.0135 (18)	0.0290 (17)	0.0006 (16)
C2	0.0447 (17)	0.059 (2)	0.052 (2)	−0.0061 (17)	0.0069 (15)	0.0015 (17)
C15	0.072 (2)	0.0405 (19)	0.0551 (19)	−0.0057 (18)	0.0306 (17)	−0.0086 (16)
C28	0.067 (2)	0.051 (2)	0.0523 (19)	−0.0091 (19)	0.0234 (17)	−0.0034 (17)
C18	0.0571 (19)	0.062 (2)	0.0465 (18)	−0.0045 (18)	0.0215 (15)	−0.0113 (17)
C20	0.0428 (17)	0.087 (3)	0.0330 (16)	−0.002 (2)	0.0141 (13)	0.0087 (18)
C19	0.0536 (19)	0.091 (3)	0.0381 (17)	−0.003 (2)	0.0211 (15)	−0.0135 (19)
C10	0.0425 (18)	0.078 (3)	0.056 (2)	0.000 (2)	0.0001 (15)	−0.009 (2)
C8	0.064 (2)	0.059 (2)	0.064 (2)	−0.014 (2)	0.0204 (18)	0.0081 (19)
C9	0.0455 (19)	0.070 (3)	0.074 (2)	−0.0159 (19)	0.0153 (18)	−0.012 (2)
C27	0.050 (2)	0.115 (3)	0.058 (2)	0.007 (2)	0.0118 (17)	0.004 (2)
C29	0.059 (2)	0.100 (3)	0.068 (2)	−0.005 (2)	0.0079 (19)	−0.018 (2)

### 6-Bromo-9,9-diethyl-N,N-diphenylfluoren-2-amine Geometric parameters (Å, º)

**Table:**

Br1—C20	1.896 (3)	C13—C14	1.371 (4)
N1—C6	1.397 (4)	C26—C27	1.509 (5)
N1—C12	1.413 (4)	C26—H30A	0.9700
N1—C13	1.431 (3)	C26—H30B	0.9700
C6—C1	1.381 (4)	C11—C10	1.367 (4)
C6—C5	1.383 (4)	C11—H26	0.9300
C12—C11	1.371 (4)	C3—C2	1.370 (4)
C12—C7	1.380 (4)	C3—H18	0.9300
C5—C4	1.369 (4)	C14—C15	1.387 (4)
C5—H16	0.9300	C14—H13	0.9300
C25—C24	1.376 (4)	C2—H19	0.9300
C25—C13	1.385 (4)	C15—H12	0.9300
C25—H2	0.9300	C28—C29	1.491 (5)
C1—C2	1.363 (4)	C28—H28A	0.9700
C1—H20	0.9300	C28—H28B	0.9700
C24—C16	1.385 (4)	C18—C19	1.384 (4)
C24—C23	1.517 (4)	C18—H9	0.9300
C16—C15	1.375 (4)	C20—C19	1.365 (5)
C16—C17	1.466 (4)	C19—H8	0.9300
C4—C3	1.373 (4)	C10—C9	1.359 (5)
C4—H17	0.9300	C10—H25	0.9300
C22—C21	1.378 (4)	C8—C9	1.360 (5)
C22—C17	1.391 (4)	C8—H23	0.9300
C22—C23	1.522 (4)	C9—H24	0.9300
C17—C18	1.380 (4)	C27—H31A	0.9600
C23—C28	1.527 (4)	C27—H31B	0.9600
C23—C26	1.536 (4)	C27—H31C	0.9600
C21—C20	1.372 (4)	C29—H29A	0.9600
C21—H6	0.9300	C29—H29B	0.9600
C7—C8	1.363 (4)	C29—H29C	0.9600
C7—H22	0.9300		
			
C6—N1—C12	122.5 (2)	H30A—C26—H30B	107.5
C6—N1—C13	119.9 (2)	C10—C11—C12	120.3 (3)
C12—N1—C13	117.5 (2)	C10—C11—H26	119.8
C1—C6—C5	118.4 (3)	C12—C11—H26	119.8
C1—C6—N1	120.5 (2)	C2—C3—C4	119.0 (3)
C5—C6—N1	121.1 (3)	C2—C3—H18	120.5
C11—C12—C7	118.5 (3)	C4—C3—H18	120.5
C11—C12—N1	119.4 (3)	C13—C14—C15	120.7 (3)
C7—C12—N1	122.1 (3)	C13—C14—H13	119.6
C4—C5—C6	120.3 (3)	C15—C14—H13	119.6
C4—C5—H16	119.9	C1—C2—C3	120.6 (3)
C6—C5—H16	119.9	C1—C2—H19	119.7
C24—C25—C13	118.8 (3)	C3—C2—H19	119.7
C24—C25—H2	120.6	C16—C15—C14	118.7 (3)
C13—C25—H2	120.6	C16—C15—H12	120.6
C2—C1—C6	120.8 (3)	C14—C15—H12	120.6
C2—C1—H20	119.6	C29—C28—C23	115.4 (3)
C6—C1—H20	119.6	C29—C28—H28A	108.4
C25—C24—C16	120.6 (3)	C23—C28—H28A	108.4
C25—C24—C23	128.0 (3)	C29—C28—H28B	108.4
C16—C24—C23	111.4 (2)	C23—C28—H28B	108.4
C15—C16—C24	120.6 (3)	H28A—C28—H28B	107.5
C15—C16—C17	130.9 (3)	C17—C18—C19	118.5 (3)
C24—C16—C17	108.5 (3)	C17—C18—H9	120.7
C5—C4—C3	120.8 (3)	C19—C18—H9	120.7
C5—C4—H17	119.6	C19—C20—C21	122.4 (3)
C3—C4—H17	119.6	C19—C20—Br1	118.7 (3)
C21—C22—C17	120.3 (3)	C21—C20—Br1	118.9 (3)
C21—C22—C23	128.5 (3)	C20—C19—C18	119.9 (3)
C17—C22—C23	111.2 (2)	C20—C19—H8	120.0
C18—C17—C22	120.8 (3)	C18—C19—H8	120.0
C18—C17—C16	131.0 (3)	C9—C10—C11	121.0 (3)
C22—C17—C16	108.2 (3)	C9—C10—H25	119.5
C24—C23—C22	100.7 (2)	C11—C10—H25	119.5
C24—C23—C28	112.0 (2)	C9—C8—C7	121.2 (3)
C22—C23—C28	113.3 (2)	C9—C8—H23	119.4
C24—C23—C26	111.2 (2)	C7—C8—H23	119.4
C22—C23—C26	110.8 (2)	C10—C9—C8	118.8 (3)
C28—C23—C26	108.6 (3)	C10—C9—H24	120.6
C20—C21—C22	118.0 (3)	C8—C9—H24	120.6
C20—C21—H6	121.0	C26—C27—H31A	109.5
C22—C21—H6	121.0	C26—C27—H31B	109.5
C8—C7—C12	120.1 (3)	H31A—C27—H31B	109.5
C8—C7—H22	119.9	C26—C27—H31C	109.5
C12—C7—H22	119.9	H31A—C27—H31C	109.5
C14—C13—C25	120.6 (3)	H31B—C27—H31C	109.5
C14—C13—N1	119.3 (3)	C28—C29—H29A	109.5
C25—C13—N1	120.0 (3)	C28—C29—H29B	109.5
C27—C26—C23	115.1 (3)	H29A—C29—H29B	109.5
C27—C26—H30A	108.5	C28—C29—H29C	109.5
C23—C26—H30A	108.5	H29A—C29—H29C	109.5
C27—C26—H30B	108.5	H29B—C29—H29C	109.5
C23—C26—H30B	108.5		
			
C12—N1—C6—C1	158.9 (3)	C17—C22—C23—C26	−115.5 (3)
C13—N1—C6—C1	−16.4 (4)	C17—C22—C21—C20	1.5 (4)
C12—N1—C6—C5	−22.9 (4)	C23—C22—C21—C20	−177.1 (3)
C13—N1—C6—C5	161.8 (3)	C11—C12—C7—C8	−0.7 (5)
C6—N1—C12—C11	133.9 (3)	N1—C12—C7—C8	−179.7 (3)
C13—N1—C12—C11	−50.7 (4)	C24—C25—C13—C14	−1.6 (4)
C6—N1—C12—C7	−47.1 (4)	C24—C25—C13—N1	176.6 (3)
C13—N1—C12—C7	128.2 (3)	C6—N1—C13—C14	118.5 (3)
C1—C6—C5—C4	−1.4 (5)	C12—N1—C13—C14	−57.0 (4)
N1—C6—C5—C4	−179.6 (3)	C6—N1—C13—C25	−59.7 (4)
C5—C6—C1—C2	1.3 (5)	C12—N1—C13—C25	124.8 (3)
N1—C6—C1—C2	179.5 (3)	C24—C23—C26—C27	−57.7 (3)
C13—C25—C24—C16	1.0 (4)	C22—C23—C26—C27	53.5 (4)
C13—C25—C24—C23	−178.2 (3)	C28—C23—C26—C27	178.6 (3)
C25—C24—C16—C15	0.4 (4)	C7—C12—C11—C10	0.3 (5)
C23—C24—C16—C15	179.7 (3)	N1—C12—C11—C10	179.3 (3)
C25—C24—C16—C17	−178.8 (3)	C5—C4—C3—C2	0.6 (5)
C23—C24—C16—C17	0.5 (3)	C25—C13—C14—C15	0.7 (5)
C6—C5—C4—C3	0.5 (5)	N1—C13—C14—C15	−177.5 (3)
C21—C22—C17—C18	−1.8 (5)	C6—C1—C2—C3	−0.2 (5)
C23—C22—C17—C18	177.1 (3)	C4—C3—C2—C1	−0.7 (5)
C21—C22—C17—C16	179.0 (3)	C24—C16—C15—C14	−1.2 (5)
C23—C22—C17—C16	−2.1 (3)	C17—C16—C15—C14	177.7 (3)
C15—C16—C17—C18	2.8 (6)	C13—C14—C15—C16	0.7 (5)
C24—C16—C17—C18	−178.1 (3)	C24—C23—C28—C29	52.8 (4)
C15—C16—C17—C22	−178.1 (3)	C22—C23—C28—C29	−60.3 (4)
C24—C16—C17—C22	1.0 (3)	C26—C23—C28—C29	176.1 (3)
C25—C24—C23—C22	177.6 (3)	C22—C17—C18—C19	0.8 (5)
C16—C24—C23—C22	−1.7 (3)	C16—C17—C18—C19	179.8 (3)
C25—C24—C23—C28	56.9 (4)	C22—C21—C20—C19	−0.3 (5)
C16—C24—C23—C28	−122.4 (3)	C22—C21—C20—Br1	−179.6 (2)
C25—C24—C23—C26	−64.9 (4)	C21—C20—C19—C18	−0.7 (5)
C16—C24—C23—C26	115.8 (3)	Br1—C20—C19—C18	178.6 (2)
C21—C22—C23—C24	−178.9 (3)	C17—C18—C19—C20	0.5 (5)
C17—C22—C23—C24	2.3 (3)	C12—C11—C10—C9	0.4 (5)
C21—C22—C23—C28	−59.1 (4)	C12—C7—C8—C9	0.4 (5)
C17—C22—C23—C28	122.1 (3)	C11—C10—C9—C8	−0.8 (5)
C21—C22—C23—C26	63.3 (4)	C7—C8—C9—C10	0.4 (6)

### 6-Bromo-9,9-diethyl-N,N-diphenylfluoren-2-amine Hydrogen-bond geometry (Å, º)

*Cg*5 is the centroid of the C17–C22 ring.

**Table:**

*D*—H···*A*	*D*—H	H···*A*	*D*···*A*	*D*—H···*A*
C2—H19···*Cg*5^i^	0.93	2.87	3.692 (4)	148

Symmetry code: (i) −*x*+2, −*y*+1, −*z*+1.

## References

[bb1] Agilent (2012). *CrysAlis PRO* and *CrysAlis RED*. Agilent Technologies Ltd, Yarnton, England.

[bb2] Bugenhagen, B., Al Jasem, Y., Al-Azani, M. & Thiemann, T. (2014). *Acta Cryst.* E**70**, o265. 10.1107/S1600536814002669 PMC3998413 24764980

[bb3] Crundwell, G., Glagovich, N. M., Heinrich, E. M. R. & Ouellette, P. (2019). *IUCrData*, **4**, x190553.

[bb4] Dufresne, S., Skalski, T. & Skene, W. G. (2011). *Can. J. Chem.* **89**, 173–180.

[bb5] Eakins, G. L., Cooper, M. W., Gerasimchuk, N. N., Phillips, T. J., Breyfogle, B. E. & Stearman, C. J. (2013). *Can. J. Chem.* **91**, 1059–1071.

[bb6] Evans, P., Izod, K. & Waddell, P. G. (2016). Private communication (refcode CCDC 1488084). CCDC, Cambridge, England.

[bb7] Groom, C. R., Bruno, I. J., Lightfoot, M. P. & Ward, S. C. (2016). *Acta Cryst.* B**72**, 171–179. 10.1107/S2052520616003954 PMC4822653 27048719

[bb8] Justin Thomas, K. R., Velusamy, M., Lin, J. T., Chuen, C.-H. & Tao, Y.-T. (2005). *Chem. Mater.* **17**, 1860–1866.

[bb9] Kent, G. T., Cook, A. W., Damon, P. L., Lewis, R. A., Wu, G. & Hayton, T. W. (2021). *Inorg. Chem.* **60**, 4996–5004. 10.1021/acs.inorgchem.1c00052 33764048

[bb10] Melen, R. L., Eisler, D. J., Hewitt, R. A. & Rawson, J. M. (2013). *Dalton Trans.* **42**, 3888–3895. 10.1039/c2dt32878j 23325301

[bb11] Seidel, P., Schwarzer, A. & Mazik, M. (2021). *Acta Cryst.* E**77**, 1029–1032. 10.1107/S2056989021009464 PMC8491529 34667632

[bb12] Sheldrick, G. M. (2015*a*). *Acta Cryst.* A**71**, 3–8.

[bb13] Sheldrick, G. M. (2015*b*). *Acta Cryst.* C**71**, 3–8.

[bb14] Spek, A. L. (2020). *Acta Cryst.* E**76**, 1–11. 10.1107/S2056989019016244 PMC6944088 31921444

[bb15] Sprachmann, J., Grabicki, N., Möckel, A., Maltitz, J., Monroy, J. R., Smales, G. J. & Dumele, O. (2023). *Chem. Commun.* **59**, 13639–13642. 10.1039/d3cc03735e 37905422

[bb16] Zhang, X.-Y., Liu, B.-N., Wang, P.-B. & Liu, D.-K. (2014). *Acta Cryst.* E**70**, o1118–o1119. 10.1107/S1600536814019564 PMC4257222 25484705

